# CAREGIVING SURVEILLANCE AT THE CDC: PAST, PRESENT, AND FUTURE

**DOI:** 10.1093/geroni/igz038.672

**Published:** 2019-11-08

**Authors:** Valerie J Edwards, C Grace Whiting

**Affiliations:** 1 Centers for Disease Control and Prevention, Atlanta, GA, Antigua and Barbuda; 2 National Alliance for Caregiving, Bethesda, Maryland, United States

## Abstract

As the U.S. population ages, caregiving has emerged as an important public health issue affecting an increasing proportion of American families. In 2015, an estimated 17.7 million people provided assistance to family members and friends. Although caregiving can have positive aspects, many studies have found that caregivers report more health difficulties than non-caregivers. The importance of population-based information is central to public health’s ability to respond effectively to this growing public health problem. The Alzheimer’s Disease and Healthy Aging Program at the Centers for Disease Control and Prevention (CDC) has made surveillance of caregivers a priority area. To this end, the development and use of a caregiving module for the Behavioral Risk Factor Surveillance System (BRFSS) was undertaken. The BRFSS is one of the largest telephone-based health surveillance system in the world, and collects information from the public across a broad range of health topics. This platform therefore provides a unique opportunity to capture health status data from caregivers as well as the option of comparing caregivers to non-caregivers. The Caregiver Module consists of 9 questions that address the characteristics of care and the type of assistance provided. The objective of this symposium is to describe the development of the current caregiving module (Dr. Bouldin), present relevant findings from the previous three years of surveillance data (Drs. Edwards and Taylor), and to discuss future directions for caregiver surveillance and CDC-developed resources to facilitate date utilization (Dr. McGuire). The discussant will describe the impact and status of national-level surveillance data

